# Identification of the Extradural and Intradural Extension of Pituitary Adenomas to the Suprasellar Region: Classification, Surgical Strategies, and Outcomes

**DOI:** 10.3389/fonc.2021.723513

**Published:** 2021-07-20

**Authors:** YouQing Yang, YouYuan Bao, ShenHao Xie, Bin Tang, Xiao Wu, Le Yang, Jie Wu, Han Ding, ShaoYang Li, SuYue Zheng, Tao Hong

**Affiliations:** Department of Neurosurgery, The First Affiliated Hospital of Nanchang University, Nanchang, China

**Keywords:** pituitary adenoma, suprasellar extension, extradural space, intradural space, classification, diaphragma sellae

## Abstract

**Objective:**

Suprasellar pituitary adenomas (PAs) can be located in either extradural or intradural spaces, which impacts surgical strategies and outcomes. This study determined how to distinguish these two different types of PAs and analyzed their corresponding surgical strategies and outcomes.

**Methods:**

We retrospectively analyzed 389 patients who underwent surgery for PAs with suprasellar extension between 2016 to 2020 at our center. PAs were classified into two main grades according to tumor topography and their relationships to the diaphragm sellae (DS) and DS-attached residual pituitary gland (PG). Grade 1 tumors were located extradurally and further divided into grades 1a and 1b, while grade 2 tumors were located intradurally.

**Results:**

Of 389 PAs, 292 (75.1%) were surrounded by a bilayer structure formed by the DS and the residual PG and classified as grade 1a, 63 (16.2%) had lobulated or daughter tumors resulting from the thinning or absence of the residual PG and subsequently rendering the bilayer weaker were classified as Grade 1b, and the remaining 34 (8.7%) PAs that broke through the DS or traversed the diaphragmic opening and encased suprasellar neurovascular structures were classified as Grade 2. We found that the gross total removal of the suprasellar part of grade 1a, 1b, and 2 PAs decreased with grading (88.4%, 71.4%, and 61.8%, respectively). The rate of major operative complications, including cerebrospinal fluid leakage, hemorrhage, and death, increased with grading.

**Conclusions:**

It is essential to identify whether PAs with suprasellar extension are located extradurally or intradurally, which depends on whether the bilayer structure is intact. PAs with an intact bilayer structure were classified as grade 1. These were extradural and usually had good surgical outcomes and lower complications. PAs with no bilayer structure surrounding them were classified as grade 2. These were intradural, connected to the cranial cavity, and had increased surgical complications and a lower rate of gross total removal. Different surgical strategies should be adopted for extradural and intradural PAs.

## Introduction

Pituitary adenomas (PAs) account for approximately 15% of all intracranial tumors that originate in the anterior pituitary gland (1–3). As the tumor increases in size, it often grows beyond the confines of the sella turcica (4). Since the lateral and superior surfaces of the sella turcica lack bony structural support, PAs are likely to extend into the parasellar and suprasellar regions (5). In past studies, approximately 80% of the macroadenomas reported were observed to extend into the suprasellar space (6–8).

PAs that significantly extend into the suprasellar region are considered invasive on imaging (9, 10). This definition of an invasive PA, with the criteria that the tumor invades into the suprasellar region, leads to confusion in diagnosis and treatment. Some PAs that significantly extend into the suprasellar region still have a bilayer structure formed by the diaphragm sellae (DS) and the thinning residual pituitary gland after tumor removal. Thus, the tumor cavity is not connected to the intracranial cavity and it seems unreasonable to classify these as invasive PAs. Other tumors create cavities that are in direct contact with the cranial cavity after excision. These tumors often encircle vital neurovascular structures. The surgical approach to and complications of such PAs are markedly different from those with unconnected tumor cavities.

Distinguishing between the two types of PA with differential suprasellar extension (SSE) is crucial to surgical strategy, though there is currently no method to distinguish between them.

There is also still no convincing theory on how PAs extend into the suprasellar space. Some suggest that they extend through the large diaphragmatic opening (5, 11) but fail to explain how an intact DS and DS-attached residual pituitary gland can still be visible without cerebrospinal fluid (CSF) leakage after tumor resection.

The purpose of this study was to determine how to objectively identify these two different forms of PA, their differential extension into the suprasellar region, and their corresponding surgical strategies, and to present our hypotheses on how the adenomas extend into the suprasellar space.

## Materials and Methods

### Patient Selection

Included in this study were 389 patients with PAs with SSE who underwent tumor removal surgery performed by lead investigator, Hong Tao, M.D. between 2016 and 2020. Patients with recurrent tumors were excluded to avoid confounding bias. Patients without preoperative magnetic resonance imaging (MRI) or with PAs lacking SSE were also excluded.

This retrospective study was approved by the University of Nanchang Institutional Review Board. Clinical and pathological characteristics were obtained from the institutional database and medical records. All patients routinely underwent endocrine and ophthalmic examinations just before surgery and 7 days post-surgery.

### MRI Evaluation and Tumor Classification

An MRI was performed on each patient just before surgery and 3 days post-surgery using a standard 3.0-T scanner. Two independent neurosurgeons classified the adenomas with guidance from senior professors (T.H. and B.T.); any divergence was discussed and resolved.

**Figure 1:**
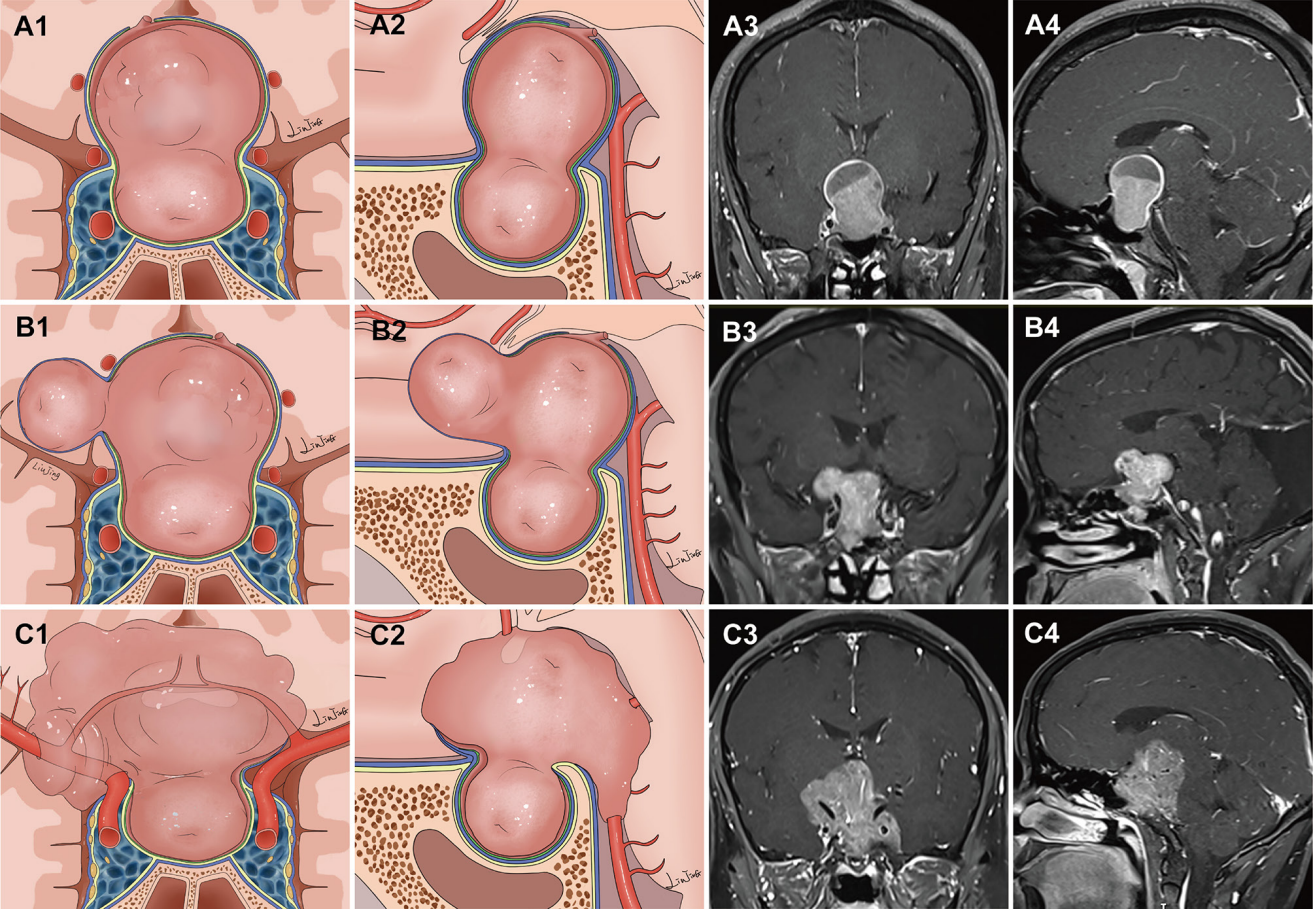
Classification of pituitary adenomas (PAs) with suprasellar extensions (SSE). Each horizontal panel represents an illustration of coronal and sagittal sectional views, and preoperative and postoperative magnetic resonance images for each type of PA. **(A1–A4)** Grade 1a PAs presented as an “inflated balloon” with expansive growth toward the suprasellar region that pushed the DS and DS-attached residual pituitary gland. **(B1–B4)** Grade 1b PAs elevated the DS and DS-attached residual pituitary gland. In some areas beneath the DS, the residual pituitary gland was compressed by the tumor, resulting in it become extremely thin or even absent. The thinning or even absence of the residual pituitary caused the bilayer to become weaker, leading to an inability to resist the intratumoral pressure. This led to the formation of daughter tumors, like a thinning tire forming a bulge. The suprasellar portion of the PAs had an asymmetrically lobulated appearance with clear border. **(C1–C4)** Grade 2 PAs broke through the DS to reach the subarachnoid space.

Tumors were classified into two main grades based on the configuration of the suprasellar portion of the tumor and its relationship to the DS, DS-attached residual pituitary gland, and major intracranial neurovascular structure. Grade 1a: PAs presented as “inflated balloons” with expansive growth toward the suprasellar region and pushed the DS and DS-attached residual pituitary gland (**Figure 1A**). The suprasellar aspects of the tumors were regularly shaped and smooth with clear borders. A thick, uneven layer of residual pituitary was often observed below the DS. The arteries of the circle of Willis were located at the edge of these tumors and still outside the DS. Grade 1b: The suprasellar portion of the adenomas had an asymmetrically lobulated appearance, with clear borders (**Figure 1B**). 1b tumors elevated the DS and its attached residual pituitary gland. In some areas beneath the DS, the residual pituitary gland was flattened by the tumor, causing an extremely thin or even absent pituitary, leading to the formation of a daughter tumor, as if a tire had thinned and formed a bulb. Blood vessels were also often located at the edges of the tumor and still outside the DS. Grade 2: The tumor broke through the DS growing into the suprasellar region. These tumors grew along the suprasellar cistern and encircled the neurovascular structures (**Figure 1C**). In these tumors, the shape of the suprasellar adenoma was irregular and matched the suprasellar cistern morphology.

The extent of tumor removal was confirmed by intraoperative findings and postoperative contrast enhanced MRI acquired within 72 hours after surgery. Gross total removal (GTR) was confirmed if no suprasellar adenoma was identified by postoperative MRI, and cases in which there were any small residual tumors were classified as subtotal resection (STR). Radical resection of the tumor from within the cavernous sinus was not involved as the focus of the study was on resection of suprasellar adenomas.

### Surgical Approach

**Figure 2:**
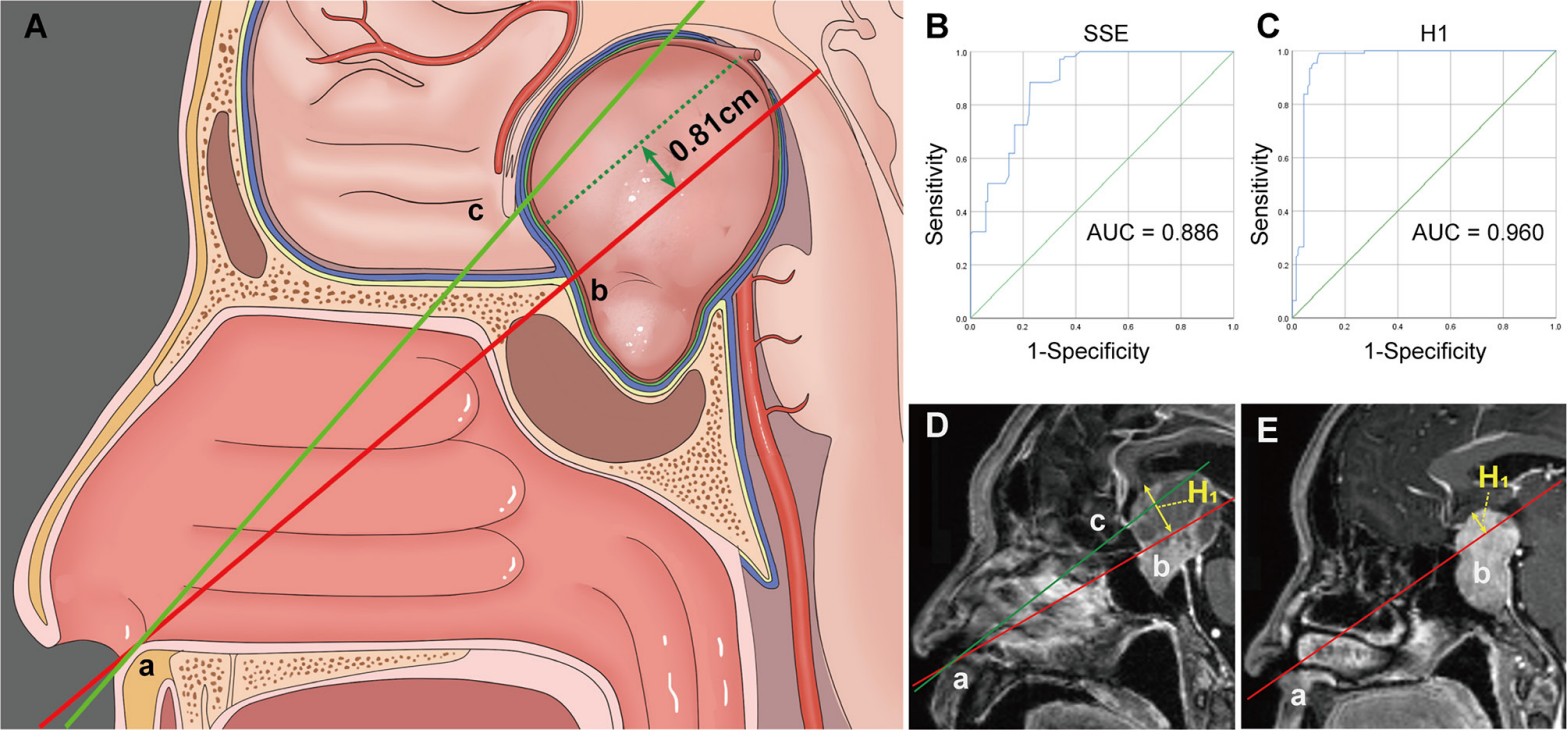
**(A)** Illustration demonstrating the “two points and one line” method. **(B, C)** Receiver operating characteristic (ROC) curves for evaluating the predictive power of the SSE **(B)** and H1 **(C)** for the expand endoscopic endonasal approach. **(D)** The vertical height (H1) from the upper surface of the tumor to the AB line is within 8.1 mm and often a standard endoscopic endonasal approach can remove the tumor. **(E)** Tumors that exceed the AB line by 8.1 mm often require an extended endoscopic endonasal approach. Point A, the inferior border of the nostril. Point B, the inferior border of the tuberculum sellae. Point C, the inferior border of the optic chiasm. H1, the vertical height from the upper surface of the tumor to the AB line.

All patients underwent surgical treatment *via* the endoscopic endonasal approach (EEA). The expanded endoscopic endonasal approach (EEEA), transsphenoidal transtuberculum approach, is a valuable treatment option for PAs with significant SSE (12–16). However, it remains unclear how deep a tumor must extend into the suprasellar region to require EEEA. Therefore, on the mid-sagittal image, the degree of SSE (17, 18) and the maximum vertical height (H1) of the upper surface of the tumor to the line joining the inferior border of the nostril and the inferior border of the tuberculum sellae were measured (**Figure 2**). A receiver operating characteristic curve (ROC) was used to evaluate the cutoff values of SSE and H1 as predictive factors for EEEA.

### Operative Technique

The standard EEA for PAs has been described previously (13, 19, 20). A 2-surgeon 4-handed technique with binostril access was employed as previously described (21, 22). Briefly, in situations where the DS descended asymmetrically, care was taken to avoid tumor residue (23). For lobulated tumors, special attention was paid to the management of the daughter tumor. Because grade 2 tumors encircled vital neurovascular structures, surgeons took particular care to avoid damaging non-tumor structures such as the perforators. After tumor removal, a multilayered reconstruction technique was performed to repair the skull base defect, as described in previous publications (24).

### Statistical Analysis

Differences between groups were assessed by Chi-square analysis (or Fisher’s exact test where appropriate). A p-value <0.05 was considered statistically significant by using SPSS software (SPSS version 25). ROC curves were generated to determine the sensitivities and specificities of the cut-off values of H1 and SSE for the prediction of EEEA.

## Results

### Patient Demographics, Tumor Characteristics, and Grading

There were 389 patients with PAs with SSEs who received surgical treatment. The mean age was 51.6 ± 13.1 years, and 52.2% (n = 203) of patients were male. The mean follow-up time was 16.2 months (range 3–54 months). Nonfunctioning PA was the most frequent tumor type (n=263, 67.4%). There were 52 (13.4%) growth hormone secreting adenomas, 34 (8.7%) prolactinomas, 12 (3.1%) ACTH secreting adenomas, 6 (1.5%) TSH-secreting adenomas, and 22 (5.7%) mixed adenomas. The prolactinomas in this study were treated surgically in patients who had either serious side effects or no reaction to a dopamine agonist. The most common clinical manifestations were visual complaints (n = 286, 73.5%) and headaches (n = 160, 41.1%), while 58 (14.9%) cases were asymptomatic (incidental detection). Anterior hypopituitarism was detected in 51 cases (13.1%). According to the criteria described above, there were 292 (75.1%), 63 (16.2%), and 34 (8.7%) patients with grade 1a, 1b, and 2 tumors, respectively (**Table 1**).

**Table 1:** Patient and tumor characteristics.

Characteristic	Value
Sex, male	203(52.2%)
Age (median ± SD [range]) (yrs)	51.6 ± 13.1 (17–79)
Suprasellar extension (According to the degree of SSE) (no. [%])	389
0mm < SSE ≤ 10mm	127 (32.6%)
10mm < SSE ≤ 20mm	149 (38.3%)
20mm < SSE ≤ 30mm	74 (19.1%)
30mm < SSE	39 (10%)
Suprasellar extension (Our suprasellar grading) (no. [%])	389
Grade 1a	292 (75.1%)
Grade 1b	63 (16.2%)
Grade 2	34 (8.7%)
Pathological types	
Nonfunctional	263 (67.6%)
GH	52 (13.4%)
PRL	34 (8.7%)
ACTH	12 (3.1%)
TSH	6 (1.5%)
Mix	22 (5.7%)

SSE, suprasellar extension; PRL, prolactin; ACTH, adrenocorticotropic hormone; GH, growth hormone; TSH, thyroid stimulating hormone.

### Surgical Outcomes

As this study focused on removal of suprasellar adenomas, radicality of tumor removal from within the cavernous sinus was not addressed. Based on the intraoperative assessment and postoperative MRI, GTR of the suprasellar adenoma was achieved in 258 (88.4%), 45 (71.4%) and 21 (61.8%) grade 1a, 1b, and 2 adenomas, respectively. The rate of GTR declined with increasing grade (*p* < 0.05) (**Table 2**).

**Table 2:** Clinical outcomes in patients with suprasellar pituitary adenomas.

	Grade 1a	Grade 1b	Grade 2	*p* Value
	(n = 292)	(n = 63)	(n = 34)	
Gross total resection, n (%)	258 (88.4)	45 (71.4)	21 (61.8)	0.004^*^
Visual dysfunction				
Improved, n (%)†	158 (73.4)	32 (71.1)	18 (69.2)	0.868
Unchanged, n (%)	130 (44.5)	29 (46)	12 (35.3)	0.553
Worsened, n (%)	4 (1.4)	2 (3.2)	3 (8.8)	0.023^*^
Postop Endocrine				
Posterior pituitary insufficiency				
Temporary DI, n (%)	30 (10.3)	8 (12.7)	4 (11.8)	0.838
Permanent DI, n (%)	13 (4.4)	3 (4.7)	2 (5.9)	0.930
New or worse anterior pituitary insufficiency, n (%)	38 (13)	9 (14.3)	5 (14.7)	0.937
CSF leak, n (%)	6 (2.1)	4 (6.3)	3 (8.8)	0.016^*^
Meningitis, n (%)	4 (1.4)	3 (4.7)	2 (5.9)	0.067
Intracranial hematoma, n (%)	5 (1.7)	3 (4.7)	4 (11.8)	0.006^*^
Intracranial ischemia, n (%)	0	0	2 (5.9)	NA
Death, n (%)	0	0	1 (2.9)	NA

DI, Diabetes insipidus; CSF, Cerebrospinal fluid.

^†^, % means “% of Pre-op abnormal.” *p < 0.05 vs. grade 1a group. All the indicators (P < 0.05) are highlighted in bold values. NA, not available.

Postoperative CSF leakage occurred in 13 (3.3%) patients: grade 1a, 6 (2.1%); grade 1b, 4 (6.3%); and grade 2, 3 (8.8%). The rate of CSF leakage was significantly higher in grade 2 than in grade 1a (*p* < 0.05) (**Table 2**).

For the patients with preoperative visual dysfunction, approximately 70% reported improvement after surgery. A total of 9 patients experienced worsening vision: grade 1a, 4 (1.4%); grade 1b, 2 (3.2%); and grade 2, 3 (8.8%). The rate of worsening vision was significantly higher in grade 2 than in grade 1a (*p* < 0.05) (**Table 2**).

Out of our cohort, 5 (1.7%) patients in grade 1a, 3 (4.7%) in grade 1b, and 4 (11.8%) in grade 2 experienced postoperative hematomas. There were no cases of intracranial ischemia or death in grade 1a and grade 1b tumors. Among those with grade 2 tumors, cerebral ischemia occurred in 2 cases and death occurred in 1 case.

### Sensitivities and Specificities of Cut-Off Values of H1 and SSE on Sagittal Views

In our cohort, 67 (22.9%) patients in grade 1a, 19 (30.2%) in grade 1b, and 25 (73.5%) in grade 2 experienced EEEA. The ROC curves for the sagittal H1 and SSE that were used to predict the need for an EEEA are shown in **Figure 2**. The area under the curve (AUC) of the ROC curve for the sagittal SSE was 0.886 (95% CI: 0.855-0.917). When the Youden index reached a maximum, the optimal cut-off value was 17.4 mm. The AUC for H1 was 0.960 (95% CI: 0.941-0.980). We found the optimal relationship between sensitivity and specificity for EEA at the cut-off value of 8.1 mm.

## Discussion

PAs with significant extension into the suprasellar region are a huge challenge for neurosurgeons. Identifying whether PAs with SSE are located extradural or intradural is essential for surgical strategies and outcomes, though there is currently no method to distinguish between them. The DS is an important barrier between the intrasellar and the suprasellar region (25–27). Hence, we established a classification system that can identify these two PAs preoperatively according to tumor topography and its relationship to the DS, DS-attached residual pituitary gland, and major intracranial neurovascular structures.

**Figure 3:**
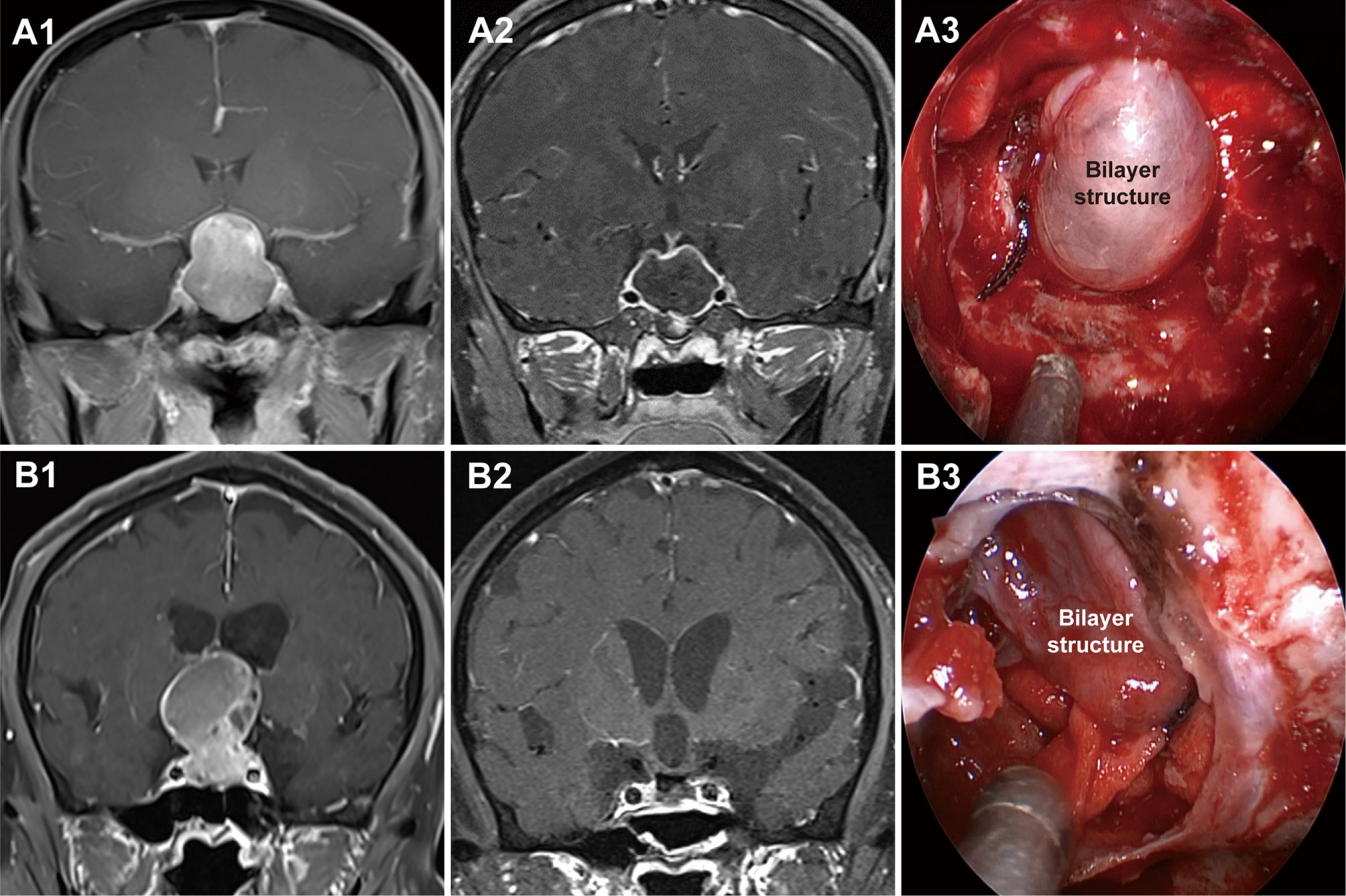
Two cases with grade 1a pituitary adenomas (PAs). **(A1, B1)** Preoperative, coronal post-gadolinium magnetic resonance image (MRI) showing grade 1a tumor with a regular smooth shape and clear border. **(A2, B2)** Postoperative MRI showing gross total resection. **(A3, B3)** Endoscopic views showing an intact bilayer structure formed by the DS and DS-attached residual pituitary gland.

Grade 1a PAs pushed and stretched the DS and the residual pituitary gland up toward the suprasellar region. Due to the restriction of the DS and DS-attached residual pituitary gland, these tumors grew expansively into the suprasellar region like an inflated balloon (**Figure 3**). The clear border and smoothly rounded superior wall of the tumors we observed suggests that the DS was intact (28). A thin, uneven layer of residual pituitary was usually observed beneath the DS (**Figure 1A3**). Intraoperatively, the intact DS and DS-attached residual pituitary gland were observed to herniate into the intrasellar space after tumor resection (**Figure 3A3**). Even in some PAs that grew significantly toward the suprasellar region, we were still able to observe an intact DS and its attached residual gland after adenomas resection (**Figure 3B3**). Therefore, we suggest that the surfaces of grade 1a suprasellar tumors are often surrounded by the DS and a thin, uneven layer of residual pituitary. The intact bilayer structure formed by the DS and the residual pituitary gland act as a barrier between the tumor and the cranial cavity, so this type of PA is located in the extradural space. Theoretically, these tumors are located in the intrasellar/subdiaphragmatic space. The bilayer structure is the key to determining whether a PA is located in the extradural or intradural region.

**Figure 4:**
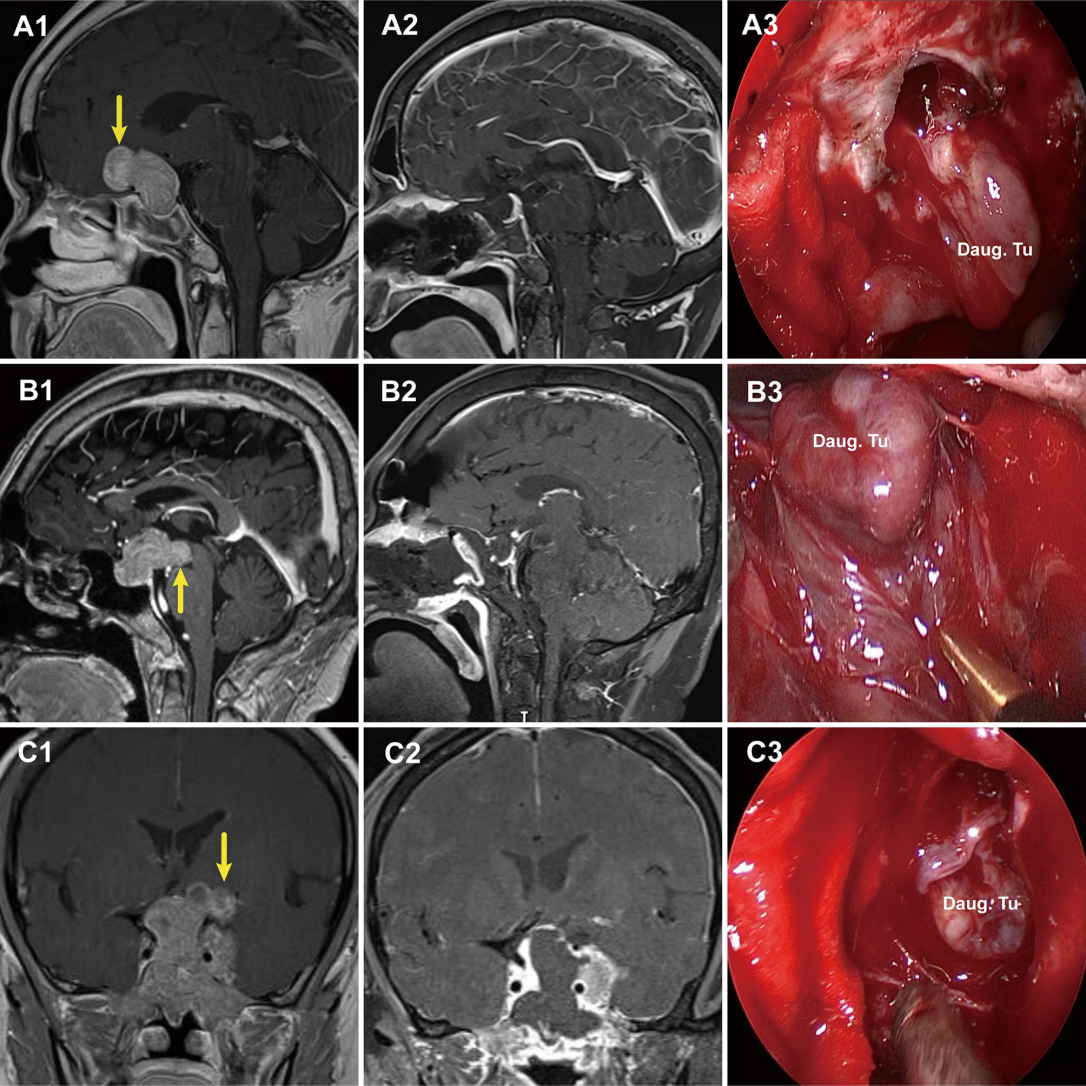
Three cases with grade 1b PAs that underwent endoscopic endonasal surgery. **(A1, B1, C1)** Preoperative, sagittal post-gadolinium MRIs showing 3 grade 1b tumors with daughter tumor (yellow arrow) extending to the anterior skull base **(A1)**, the interpeduncular fossa **(B1)**, and the suprasellar lateral **(C1)**. **(A2, B2, C2)** Postoperative MRI demonstrating gross total resection of suprasellar part of tumor was achieved. **(A3, B3, C3)** Endoscopic views showing the intact wall of daughter tumor. Daug. Tu, daughter tumor; DS, diaphragm sellae.

**Figure 5:**
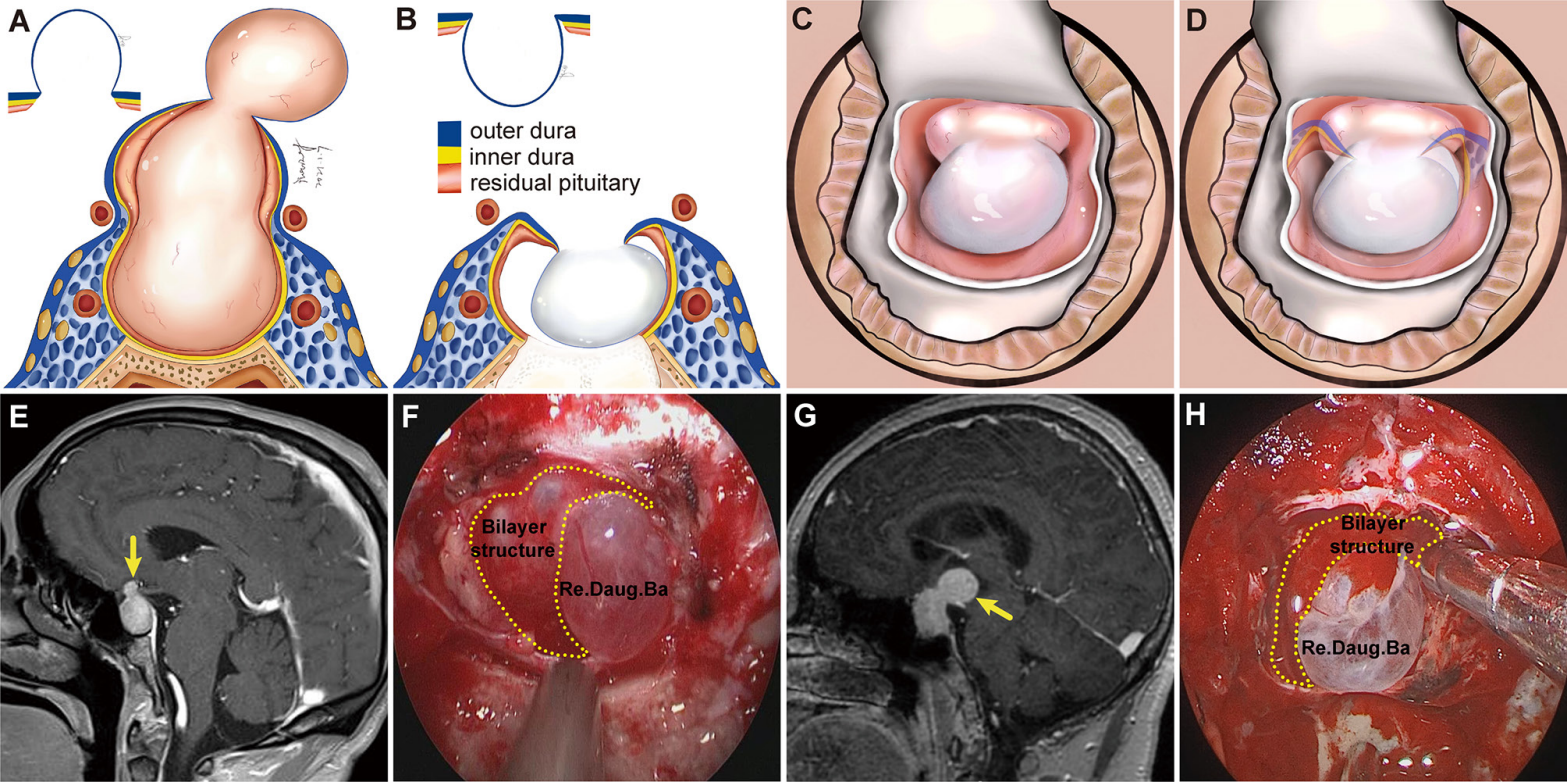
Schemes, preoperative magnetic resonance images (MRI), and endoscopic views showing a daughter tumor and reverse daughter balloon. **(A)** Illustration of the formation of a daughter tumor. The thinning or even absence of the residual pituitary caused the bilayer to be weaker, leading to an inability to resist the intratumoral pressure. Thus, a daughter tumor formed, like a thinning tire creating a bulge. **(B, C)** Illustration of reverse daughter balloon. When the tumor was completely removed, due to the intracranial cerebrospinal fluid pressure, the thin dura on the surface of the daughter tumor inversely protruded into the tumor cavity, forming a reverse daughter balloon. Diagram **(D)** is a merge of diagrams **(B, C)**. **(E, G)** Preoperative, sagittal post-gadolinium MRI showing grade 1b tumor with a small daughter tumor extending into the suprasellar space. **(F, H)** Endoscopic view after tumor resection showing a reverse daughter balloon. Re. Daug. Ba, reverse daughter balloon.

Compared to Grade 1a, Grade 1b PAs grew further and stretched the DS and DS- attached residual gland into the suprasellar region. The relatively thin residual pituitary gland beneath the DS was compressed and stretched so much by the tumor that it became extremely thin or even absent, leading to weakness in this area and an inability to resist the intratumoral pressure (**Figure 4**). This led to the formation of daughter tumors (**Figure 5A**). Despite being thin, the walls of the daughter tumors remained continuous with the DS. Thus, theoretically, these tumors were still located in the intrasellar/subdiaphragmatic space and classified as grade 1. When the area of weakness in the DS was relatively large, a wide-necked daughter tumor formed, while when the area was relatively small, a narrow-necked daughter tumor formed. Both single and multiple daughter tumors of varying sizes could be formed in the same patient. The daughter tumor could extend in the direction of the anterior cranial base, temporal lobe, and clivus (**Figures 4A1–C1**). Intraoperatively, a transparent and extremely thin wall of the daughter tumor could be observed inversely projecting into the tumor cavity forming a reverse daughter balloon after the tumor removal (**Figure 5F**), just as the DS descended into the sellar region after the tumor removal (**Figure 5**). The descending wall of reverse daughter balloon has no residual pituitary or only an extremely thin remnant pituitary attached that appears similar to the arachnoid membrane. Here, blood vessels were pushed by the tumor, and located at the tumor’s edge. In some cases, blood vessels were located at the bifurcation of multiple lobulated daughter tumors, and the bifurcation covered the vessels, similar to blood vessel being encircled.

**Figure 6:**
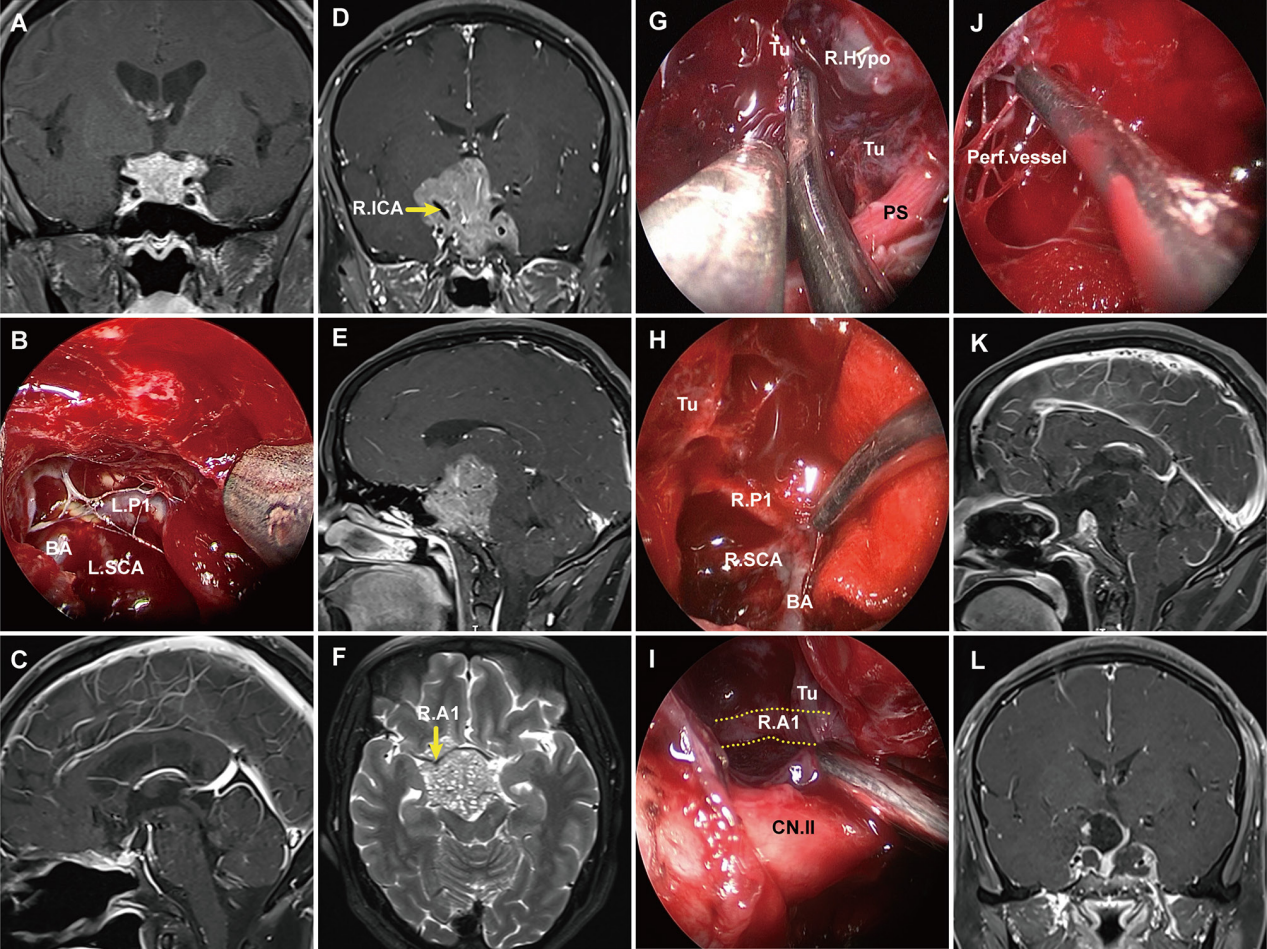
Pre- and postoperative magnetic resonance imaging (MRI) demonstrating two cases of grade 2 pituitary adenoma. **(A)** A grade 2 PA that extended to the suprasellar region and grew along with the suprasellar cistern. **(B)** Endoscopic views showing the tumor entered into the arachnoid spaces and encased vital neurovascular structures. **(C)** postoperative MRI showing gross total resection. **(D–F)** Preoperative T1 contrast-enhanced images showing a grade 2 tumor significantly extended to the suprasellar and encircled vital neurovascular structures. **(G–J)** Endoscopic views showing the tumor entered into the suprasellar space and encased vital neurovascular structures. The tumor grew in grid-like structures formed by the perforating vessels **(J)**. **(K, L)** Postoperative T1 contrast-enhanced MRI demonstrating subtotal resection of the suprasellar component of adenoma. PS, pituitary stalk; PG, pituitary gland; Tu, tumor; R. A1, right A1 segment of anterior cerebral artery; R. P1, right P1 segment of posterior cerebral artery; SCA, superior cerebellar artery; BA, basilar artery; R. Ht, right hypothalamus.

Grade 2 PAs broke through the DS or transgressed the diaphragmic opening to reach the intracranial cavity. Here, the lack of the bilayer barrier allowed the tumor to communicate directly with the cranial cavity, so this type of tumor was classified as an intradural subarachnoid tumor. Because of the lack of constraint by the bilayer, they lost their expansive growth characteristics and, like epidermoid cysts, spread along the subarachnoid space into the available space and encircled vital neurovascular structures such as the circle of Willis and its perforators, optic nerve, and optic chiasm (**Figure 6**). These tumors could even invade the floor of the third ventricle and enter the ventricular space. We have found that some PAs not extending very deep into the suprasellar cistern could also break through the DS into the intracranial cavity (**Figure 6A**). Notably, such tumors grew in grid-like structures formed by the perforating vessels (**Figure 6J**), posing a great challenge for tumor resection. These tumors could break though the Liliequist membrane and grow along the prepontine cistern into the clivus, characteristics unique to grade 2 PAs (characteristics of each grade show in **Table 3**).

**Table 3:** Characteristics and surgical strategies and outcomes of each grade.

Variable	Grade 1a	Grade 1b	Grade 2
**Location**	Located in extradural space	Still located in extradural space	Located in intradural space
**Bilayer structure (the DS and the residual pituitary gland)**	Intact	The DS was intact	Lack of a bilayer structure, directly penetrate the DS or extend through the opening of DS into the suprasellar region
		Thinning or absence of the residual pituitary gland in the area where the daughter tumor was formed	
**Location of the residual pituitary gland**	The residual pituitary gland usually located on the superior and lateral surface of the tumor	The residual pituitary gland usually located on the superior and lateral surface of the tumor and was extremely thin or even absent at the site of the daughter tumor formation	The residual pituitary gland usually located on the bottom and lateral surface of the tumor
**Relationship with vessels**	Pushed the vessels and vessels located at the edge of the tumor and still outside the DS	Pushed the vessels and vessels located at the edge of the tumor and still outside the DS	Encircled the arteries of the circle of Willis, optic nerve, and optic chiasm
**Growth pattern**	“Inflatable ball” type of spherical expansion	spherical expansion, “Tire bulge”-like formation of large or small daughter balloons	Lost expansive growth characteristics and growth along with the arachnoid cistern
**Morphology**	A regular morphology with a smooth spherical surface	An irregularly lobulated appearance with clear border	An irregular shape, and matched the morphology of the suprasellar cistern
**Surgical strategy**	Preferred EEA	Preferred EEA	When such tumors are giant, the transcranial approach can also be an appropriate choice. When the tumor is coaxial with the transsphenoidal route, the EEA may be preferred. If the tumor is giant or extends laterally to the temporal lobe with sphenoidal or cavernous sinus invasion, a combined transcranial and EEA approaches may be more appropriate.
		If the daughter tumor extended from the retro-chiasmatic region to its superior anterior aspect, significantly lateral to the suprasellar cistern, a transcranial route or transcranial combined transsphenoidal approach might be required	
**Extent of resection**	Higher rate of GTR relative to grade 1b and grade 2	Lower rate of GTR relative to grade 1a, residual tumors tended to remain in the daughter tumor	Lower rate of GTR relative to grade 1a
**Complication**	Lower	Medium, the dura mater on the surface of the daughter tumor is thin and prone to rupture leading to intraoperative CSF leakage	Higher, intraoperative CSF leak occurs 100% and is prone to serious complications such as neurovascular injury, cerebral hemorrhage, cerebral ischemia and hypothalamic injury

DS, diaphragm sellae; EEA, endoscopic endonasal approach; CSF, cerebrospinal fluid; GTR, Gross total removal.

Surgical approaches to resection were determined by tumor grade. Grade 1a PAs were located in the extradural/subdiaphragmatic space. The transsphenoidal approach is more appropriate for grade 1a PAs. However, we preferred EEA, owing to its advantages including wide-angle view and elimination of brain retraction (12, 14, 29–31). Special attention should be given to protect the residual pituitary gland intraoperatively. When the residual pituitary is observed, the boundary of the tumor has been reached. If the tumor is removed and only a thin, transparent DS remains, the residual pituitary gland might have been excised. Thus, establishing the concept of a bilayer separating the cranial cavity from the tumor facilitates the protection of the residual pituitary gland. The transsphenoidal approach was also appropriate for grade 1b PAs, as they were also located in the extradural/subdiaphragmatic space. We utilized an EEA, which allowed closer visualization of the daughter tumor. Some tumor locations were difficult to reach with EEA (12, 13, 32, 33), requiring a transcranial route or transcranial combined transsphenoidal approach. For example, this approach was used when the daughter tumor extended from the retro-chiasmatic region to its superior anterior aspect, significantly lateral to the suprasellar cistern. Special attention should be paid to the treatment of daughter tumors intraoperatively. With EEA, when the neck of the daughter tumor was wide, the tumor could be resected along the natural corridor of the daughter tumor, using the opening of the daughter tumor as a starting point to remove the tumor within the daughter tumor. when the neck of the daughter tumor was small, the neck opening could be widened to remove the tumor within the daughter tumor. When the tumor within the daughter tumor was completely removed, because of the intracranial cerebrospinal fluid pressure, the thin dura on the surface of the daughter tumor could inversely protrude into the tumor cavity. Forming a reverse daughter balloon was a sign of complete removal of the daughter tumor.

The transsphenoidal and transcranial approaches were all very difficult for grade 2 PAs, as these tumors were located in the intradural space. These tumors grew in grid-like structures formed by the perforating vessels, and it was necessary to both remove the tumor and avoid damaging the perforators. In some patients, the third ventricle floor was compressed into a thin layer, and protection of the floor of the third ventricle is necessary to avoid damage to the hypothalamus. For these reasons, when such tumors are giant, the transcranial approach can also be an appropriate choice. When the tumor is coaxial with the transsphenoidal route, EEA may be preferred. If the tumor is giant or extends laterally to the temporal lobe with sphenoidal or cavernous sinus invasion, a combined transcranial and EEA approaches may be more appropriate.

We used the two-point-one-line method to predict the need for EEEA. Our data show that both SSE and H1 are effective predictors of the need for EEEA. The cut-off value of SSE was 17.4 mm, so Hardy’s classification grades C and D may necessitate an EEEA. H1 can reflect the degree of tumor extension to the anterior skull base. We suggest that H1 is a more appropriate predictor of needing an EEEA. An EEEA is usually required for grade 1b, grade 2, and large or hard consistency grade 1a PAs.

The goal of the surgery was optic apparatus decompression and the safest possible tumor resection (32, 34). The GRT of suprasellar tumor in grade 1a PAs was higher than that of grade 1b and grade 2 tumors, due to the regular shape and the protection of the bilayer structure. Some residual tumor tended to remain in the daughter tumor, so the total resection rate was significantly lower than that of grade 1a PAs. Grade 2 tumors encircled vital blood vessels, making them more difficult to remove. Some grade 2 PAs were close to the hypothalamus, and care was taken to protect the hypothalamus and the floor of the third ventricle. These are among the reasons for incomplete resection in our series.

Severe postoperative complications, including postoperative hemorrhage, visual deterioration, cerebral infarction, and death occurred, though mostly in patients with grade 2 tumors. We believe that grade 2 tumors encircled vital neurovascular structures and were in close proximity to the hypothalamus, which greatly increased the chance of incidental damage during surgery. The tumors grew in the grids formed by the penetrating vessels, particularly in sites with abundant perforator vessels. Resection of tumors in these grids was prone to serious complications, such as postoperative stroke, due to damage to the perforator vessels.

The dura mater on the surface of daughter tumors was very thin and easily ruptured, potentially increasing the rate of CSF leakage. In our data, the rates of CSF leakage were higher in patients with grade 1b than grade 1a tumors, but not significantly. Since grade 2 PAs broke through the DS and extended into the subarachnoid space, the CSF leakage rate was significantly higher than that of patients with grade 1a PAs.

We suggest a possible pattern in which PAs extend into the suprasellar region. In grade 1 PAs, the tumor may originate in the lower part of the pituitary gland and push and stretch the pituitary gland and diaphragmatic dura upward into the suprasellar space. The possible patterns in which grade 2 PAs break through the DS into the suprasellar region include: (1) A grade 1b tumor further grows and traverses the extremely thin layer of the daughter tumor into the subarachnoid space. The distribution of the residual pituitary glands in grade 2 PAs formed this way are the same as that of grade 1b PAs. (2) In the early stages of PA growth, the tumor directly invades and penetrates the DS into the suprasellar region; (3) The tumor grows into the intracranial space through the loose diaphragmatic opening. For the latter two situations, the tumor may originate near the upper surface of the pituitary gland and easily transgress the DS or diaphragmatic opening. We have made these assumptions based on the location of the residual pituitary in these tumors. For example, the residual pituitary gland of grade 1 tumors tends to be located on the superior and lateral surface of the tumor. In contrast, the residual pituitary gland of grade 2 tumors (except those developed from grade 1b) tends to be located on the bottom and lateral surface of the tumor.

Goel et al. divided giant pituitary adenomas into 4 grades depending on their anatomical extensions and the nature of their meningeal coverings (28). In our classification, grade 1 PAs are beneath the DS, which is consistent with Goel’s classification of grade I. However, we suggest that it is not only the DS that was elevated by the tumor but also the residual pituitary gland. The surfaces of these tumors were covered by an intact bilayer structure formed by the DS and the residual pituitary. We propose that the thinning or absence of the residual pituitary gland caused the double barrier (DS and residual pituitary) to become a weak barrier, which was the key to the formation of daughter tumors.

This study has some limitations. First, our classification focused on identifying whether PAs with SSEs are located in extradural or intradural spaces, but did not take into account the degree of the suprasellar and lateral tumor extensions. In the future, we hope to reconcile our study with Hardy’s classification. In grade 1b and 2 tumors, for instance, if the tumor extends significantly lateral to the suprasellar region, a Hardy’s classification grade D, a transcranial approach or a combined transsphenoidal approach may be more appropriate. Another limitation was that our proposed two-point-one-line method of predicting when an EEEA was required is only a prediction. As such, the need for an EEA may also be related to other factors, particularly the consistency of the tumor, revision surgery, and intraoperative CSF release.

## Conclusion

The bilayer structure formed by the DS and the residual pituitary gland is the key to determining whether a PA is located in the extradural or intradural space. The grade 1 PAs are located extradural which we further divided into 2 subtypes: grade 1a and grade 1b PAs. Grade 1a PAs push and stretch the bilayer structure up toward the suprasellar region. Grade 1b PAs demonstrate a thinning or absence of residual pituitary gland, leading to weakness of the bilayer structure and formation of a daughter tumor. Grade 2 PAs have a surface that is not surrounded by a bilayer structure and are located in the intradural space. GTR decreases with increasing grade, while the surgical risks and complications increase with increasing grade. Given the information provided by this classification scheme, surgeons can plan the most appropriate operative approach and extent of resection and can better predict the surgical outcomes.

## Data Availability Statement

The original contributions presented in the study are included in the article/supplementary material. Further inquiries can be directed to the corresponding authors.

## Ethics Statement

The studies involving human participants were reviewed and approved by Institutional Ethics Committee of the First Affiliated Hospital of Nanchang University. Written informed consent to participate in this study was provided by the participants’ legal guardian/next of kin. Written informed consent was obtained from the individual(s), and minor(s)’ legal guardian/next of kin, for the publication of any potentially identifiable images or data included in this article.

## Author Contributions

TH and YY conceived of and directed the study. TH, YB, SX, and BT provided clinical information and analysis the data. XW, LY, and JW collected the data. SL and SZ made the tables and figures. TH, YY, YB, and SX reviewed the manuscript. All authors contributed to the article and approved the submitted version.

## Funding

This study was supported by the National Natural Science Foundation of China (grant nos. 82060246 and 81460381), the Ganpo555 Engineering Excellence of Jiangxi Science and Technology Department (2013), and the Key Research and Invention Plan of Jiangxi Science and Technology Department (20192BBG70026).

## Conflict of Interest

The authors declare that the research was conducted in the absence of any commercial or financial relationships that could be construed as a potential conflict of interest.
